# The placental specific gene, PLAC1, is induced by the Epstein-Barr virus and is expressed in human tumor cells

**DOI:** 10.1186/1743-422X-11-107

**Published:** 2014-06-09

**Authors:** Xia Wang, Melody C Baddoo, Qinyan Yin

**Affiliations:** 1 Section of Pulmonary Diseases, Critical Care and Environmental Medicine, Department of Medicine, SL9, Tulane University School of Medicine, 1430 Tulane Avenue, New Orleans, LA 70112, USA; 2 Department of Pathology, SL79, Tulane University School of Medicine, 1430 Tulane Avenue, New Orleans, LA 70112, USA

**Keywords:** EBV, PLAC1, Cancer, Breast, Ovarian, Prostate

## Abstract

**Background:**

The Epstein-Barr virus (EBV) is a causal agent in a number of malignancies in humans including hematopoietic tumors and non-hematopoietic tumors. Burkitt’s lymphoma cell lines containing the Epstein-Barr virus have been shown to form tumors in nude mice while clonal derivatives of such cell lines in which the viral genome has been lost do not (JID 177: 1194-1201, 1998; JV 72: 9150-9156, 1998; JV 68: 6069-6073, 1994). The re-introduction of EBV into these EBV negative BLs reconstitutes the tumor phenotype. Thus, EBV-induced cellular genes play critical role in EBV-related tumors.

**Methods and results:**

In an attempt to identify cellular genes regulated by EBV that may contribute to its tumorigenic properties, we have enforced genome loss in the Burkitt’s lymphoma (BL) line, MutuI, by introducing a dominant negative form of the episomal replication factor, EBNA1 and carried out gene array analysis. One of the genes identified by this analysis is PLAC1, a gene originally identified as being expressed exclusively in placental tissue. Real time RT-PCR analysis verified higher expression in EBV positive vs. EBV negative Mutu clones. Analysis of a panel of RNAs from 20 normal tissues demonstrated the highest level of expression in placenta but significant expression was also observed in testis and brain cerebellum. PLAC1 expression was also observed in non-BL tumor cell lines derived from breast, ovary, and prostate. Lastly, expression of PLAC1 was found to be higher in some primary breast tumors compared to normal adjacent tissues.

**Conclusion:**

This data suggests that the EBV-induced PLAC1 is a member of the cancer/testis group of tumor antigens.

## Background

Placental specific gene 1 (*Plac1*) is a member of a family of genes that have been identified as showing predominantly placenta specific gene expression [1-5] with expression being restricted to cells of trophoblastic lineage in the mouse and human [1,2]. Trophoblasts play numerous key roles in fetal development determined, in part, by differentiating along distinct pathways. These include the controlled invasion of extravillus trophoblasts into maternal uterine tissue resulting in placental attachment and regulatory interactions with the uterine vasculature. Additionally, multinucleated syncytiotrophoblasts are metabolically active cells that are in direct contact with the maternal circulation. They are responsible for many of the functions essential to pregnancy maintenance, i.e. nutrient and oxygen transport to the fetus, hormone production and secretion, and elimination of fetal waste products. As a support mechanism for these latter functions, trophoblasts specifically promote angiogenesis in part through the production of human chorionic gonadotropin (hCG) and vascular endothelial growth factor (VEGF) [6,7]. Lastly, the placenta also contributes to immune tolerance necessary for effective placentation and pregnancy maintenance.

The PLAC1 gene is located on the X chromosome (Xq26.3) near the hypoxanthine-guanine phosphoribosyl transferase (HPRT) gene [1]. PLAC1 is a 212 amino acid protein containing a predicted amino terminal transmembrane domain and a cleavable signal peptide of 23 amino acids (with a cleavage site following amino acid 23 [1]). PLAC1 has homology to the zona pellucida 3 protein (ZP3), a sperm binding glycoprotein which confers species specificity during fertilization [1], and has a “zona pellucida sperm-binding protein signature” between amino acids 89 and 106. Analysis of RNAs from a panel of normal tissues showed predominantly placental expression. While little is known regarding the function of PLAC1, lower circulating levels of PLAC1 mRNA in maternal serum have been shown to be associated with vaginal bleeding and threatened abortion prior to week 20 suggesting a key role for PLAC1 in trophoblast function [8]. Recently, PLAC1 is recognized as an essential factor for normal placental and embryonic development in mutant mouse model [9].

The identification of tumor specific antigens for the development of possible immunological cancer vaccines has been of significant interest. The accumulated identification of tumor specific antigens led to the recognition of a wide array of genes of diverse function but sharing a number of unique characteristics [10] including expression in normal tissue being restricted to testis (and often placenta), expression in a number of cancers, association with tumor grade, and immunogenicity in cancer patients [10]. In addition, many of these genes have been found to be localized on the X chromosome. Based on these common characteristics of genes with otherwise diverse functions led to their being classified together and referred to as “cancer/testis antigens (CT antigens)” [10]. Due to its localization on the surface of cancer cells, PLAC1 provides accessibility to antibodies make them attractive candidates for targeted immunotherapeutic approaches for breast cancer and other tumor types [11].

The Epstein-Barr virus (EBV) is a causal agent in a number of malignancies in humans including nasopharyngeal carcinoma, the endemic form of Burkitt’s lymphoma (BL), Hodgkin’s disease, and a small percentage of gastric carcinomas [12,13]. EBV is especially problematic in immuno-compromised individuals and despite the utilization of highly active retroviral therapy (HAART) to treat AIDs patients, the incidence of EBV associated non-Hodgkin’s lymphomas have increased [14,15].

In most tumors, EBV exists as an autonomously replicating episome. Episomal replication and segregation during mitosis is facilitated in part by the EBV encoded nuclear antigen, EBNA1 (*E*pstein-*B*arr virus *n*uclear *a*ntigen *1*) [16,17]. Previous studies have shown that either long term culture, clonal selection, or treatment with hydroxyurea can lead to the loss of EBV episomes in EBV positive Burkitt’s lymphoma cell lines [18-20]. While these EBV negative derivatives proliferate in tissue culture, the parental BLs form colonies in soft agar and form tumors in nude mice, but the EBV negative derivatives do not. Further, the re-introduction of EBV into these EBV negative BLs reconstitutes the tumor phenotype. In an effort to identify cellular genes regulated by EBV that may contribute to the malignant phenotype, we enforced EBV genome loss in the EBV positive BL line, MutuI, and carried out gene array analysis of the resulting EBV negative clones vs. the EBV positive progenitor cells. Here we report that one of the genes identified in this analysis is PLAC1. We also show that this gene is expressed in testis and that it is expressed in other tumors not associated with EBV. Based on its expression profile and its localization to the X chromosome, we propose that PLAC1 is a candidate CT antigen.

## Results

**Figure 1:**
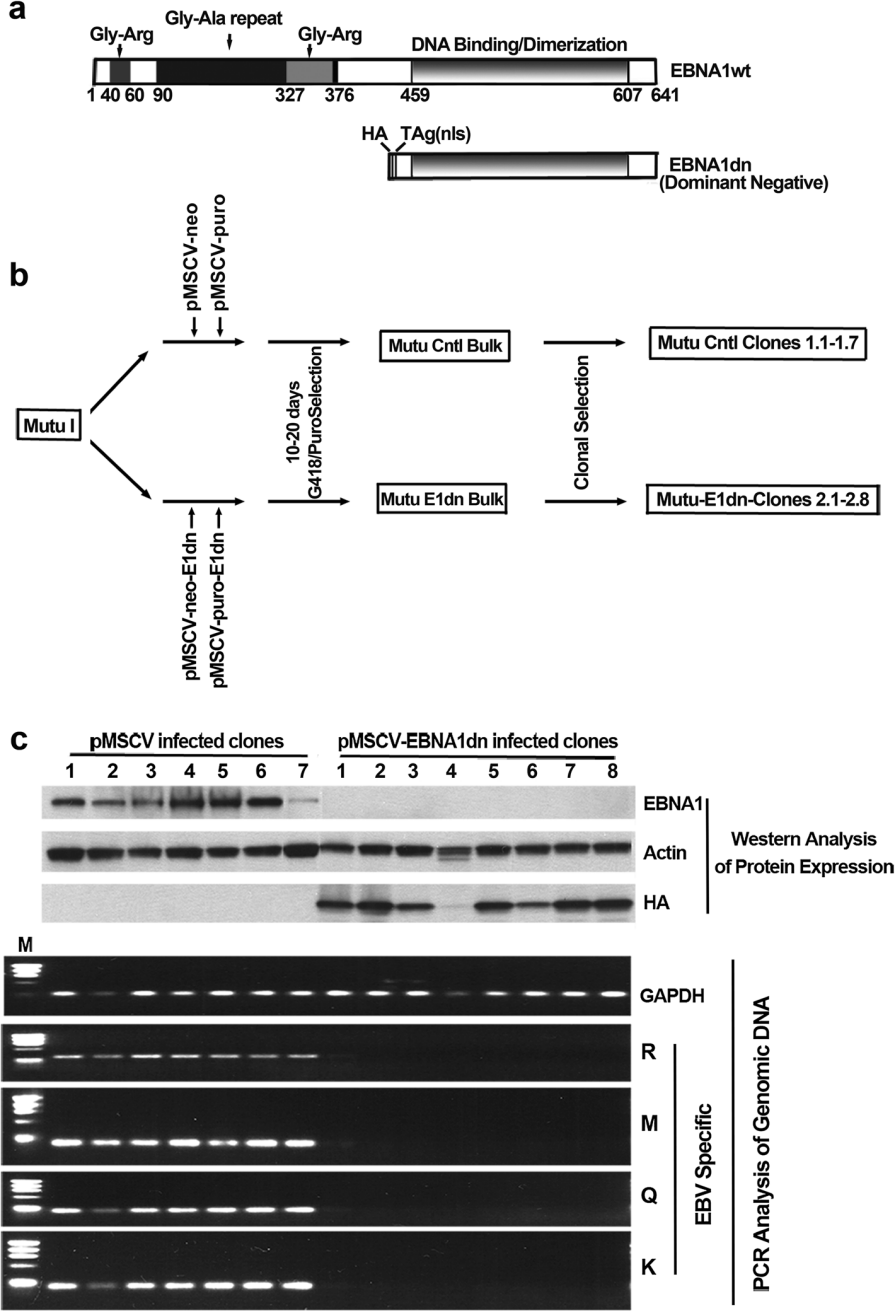
**Generation of EBV negative derivatives of MutuI cells. a)** Schematic representation of wild type and dominant negative forms of EBNA1. **b)** Scheme for infection and selection of retrovirally infected MutuI cells. **c)** Analysis of infected clones for the presence of EBV. Top panel shows Western blot analysis of EBNA1, actin and EBNA1dn. Antibodies used are described in materials and methods section. Lower panels show PCR results analyzing the presence of EBV DNA in clones. Primers were generated which amplify regions within the indicated BamHI EBV genomic fragments.

**Figure 2:**
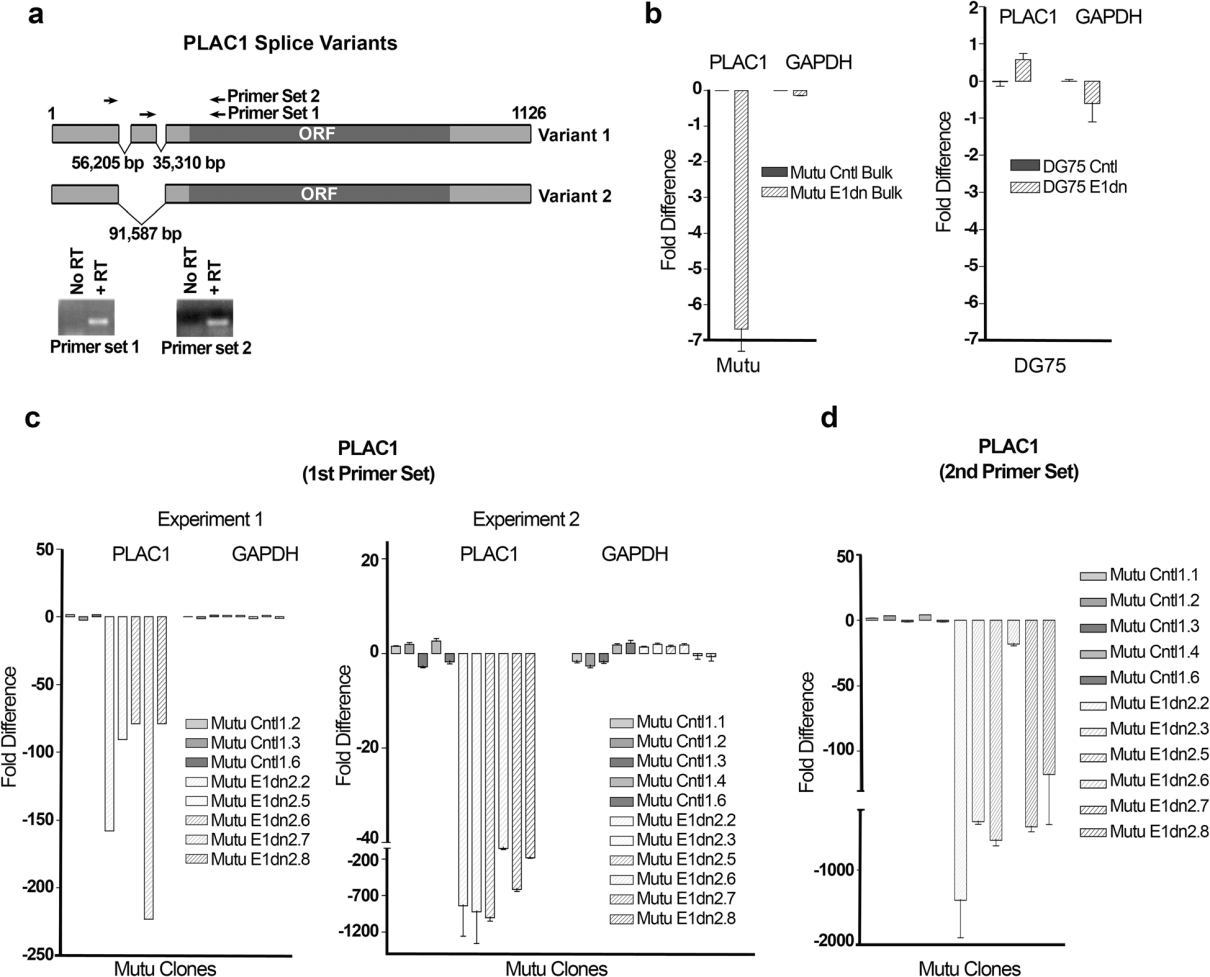
**Expression of PLAC1 in EBV positive vs. EBV negative MutuI cells. a)** Schematic representation of two splicing variants for PLAC1 and position of PCR primers used for real time RT-PCR reactions. Primers are designed to span introns to avoid any potential amplification of trace amounts of genomic DNA in RNA preparations. Gels show the amplified products from MutuI cells after 40 cycles. **b)** Real time RT-PCR analysis of RNA derived from Mutu E1dn bulk vs Mutu Cntl bulk cultures and EBNA1dn infected DG75 (EBV negative) BL cell line. **c)** Real time RT-PCR analysis of E1dn (EBV negative) and control (EBV positive) clones using primer set 1. **d)** Real time RT-PCR analysis of E1dn (EBV negative) and control (EBV positive) cells using primer set 2.

To facilitate genome loss of EBV genomes in the BL line, MutuI, a dominant negative form of the EBV episomal replication/maintenance factor, EBNA1, was generated as depicted in Figure 1a. An HA epitope tag and the nuclear localization signal from the SV40 T antigen were fused in frame to track expression and enforce nuclear localization of the resulting protein, respectively. The resulting gene cassette, EBNA1dn, was introduced into two retroviral vectors, pMSCV-neo and pMSCV-puro and introduced into MutuI cells on successive infections with a 2 day interval between infections (Figure 1b). pMSCV-neo-EBNA1dn and pMSCV-puro-EBNA1dn infected cells and control infected cells (infected with pMSCV-neo and pMSCV-puro) were selected for 10 days following which a portion of cells were frozen in liquid nitrogen and a portion were plated on 96 well plates for cloning. In some cases, the frozen bulk cultures were thawed and RNA was generated within 10 days for analysis. Notably, only limited cell death was observed indicating that the infection efficiency was relatively high and that there was not a strong selection against expression of EBNA1dn during this interval. Analysis of 8 EBNA1dn infected clones showed no detectable EBNA1 expression (Figure 1c) whereas all control infected cells showed EBNA1 expression. PCR analysis of DNA isolated from clones using primers that amplify 4 different loci of the EBV genome revealed a complete loss of EBV in clones 2–8 and trace amounts in clone 1 (Figure 1c).To identify EBV regulated genes, total RNA was isolated from clone 2.5 (EBNA1dn) and Mutu Cntl Bulk cultures and subjected to gene array analysis using an Affymetrix U133A2.0 gene chip. In a separate experiment, RNA was isolated from clone 2.7 (EBNA1dn) and again compared to freshly prepared total RNA from Mutu Cntl bulk cells. One gene identified as being expressed at a lower level in both EBNA1dn clones compared to the Cntl bulk cultures was PLAC1. Two sets of PCR primers which amplify the splice variants of PLAC1 were designed and used to analyze PLAC1 expression in control vs EBNA1dn cultures (Figure 2a). Expression of PLAC1 in Mutu E1dn bulk cultures and Mutu Cntl bulk cultures were first tested. Notably real time PCR analysis of EBV genomes showed approximately 15 fold lower number of EBV genomes in Mutu E1dn Bulk cultures compared to Mutu Cntl Bulk cultures (data not shown). Real time RT-PCR analysis showed approximately 6.5 fold lower expression of PLAC1 in the Mutu E1dn bulk cultures compared to Mutu Cntl bulk cultures (Figure 2b, left panel). In contrast, no difference in PLAC1 expression was observed in an EBV negative Burkitt’s lymphoma cell line, DG75, which was transduced with pMSCV-puro-E1dn (Figure 2b, right panel). Analysis of PLAC1 expression in control infected Mutu clones and EBNA1dn infected Mutu clones (which are devoid of EBV genomes) showed a more pronounced difference in PLAC1 expression using either primer set 1 or primer set 2 (Figure 2c and 2d). These results indicate that EBV is required for high level expression of PLAC1.

**Figure 3:**
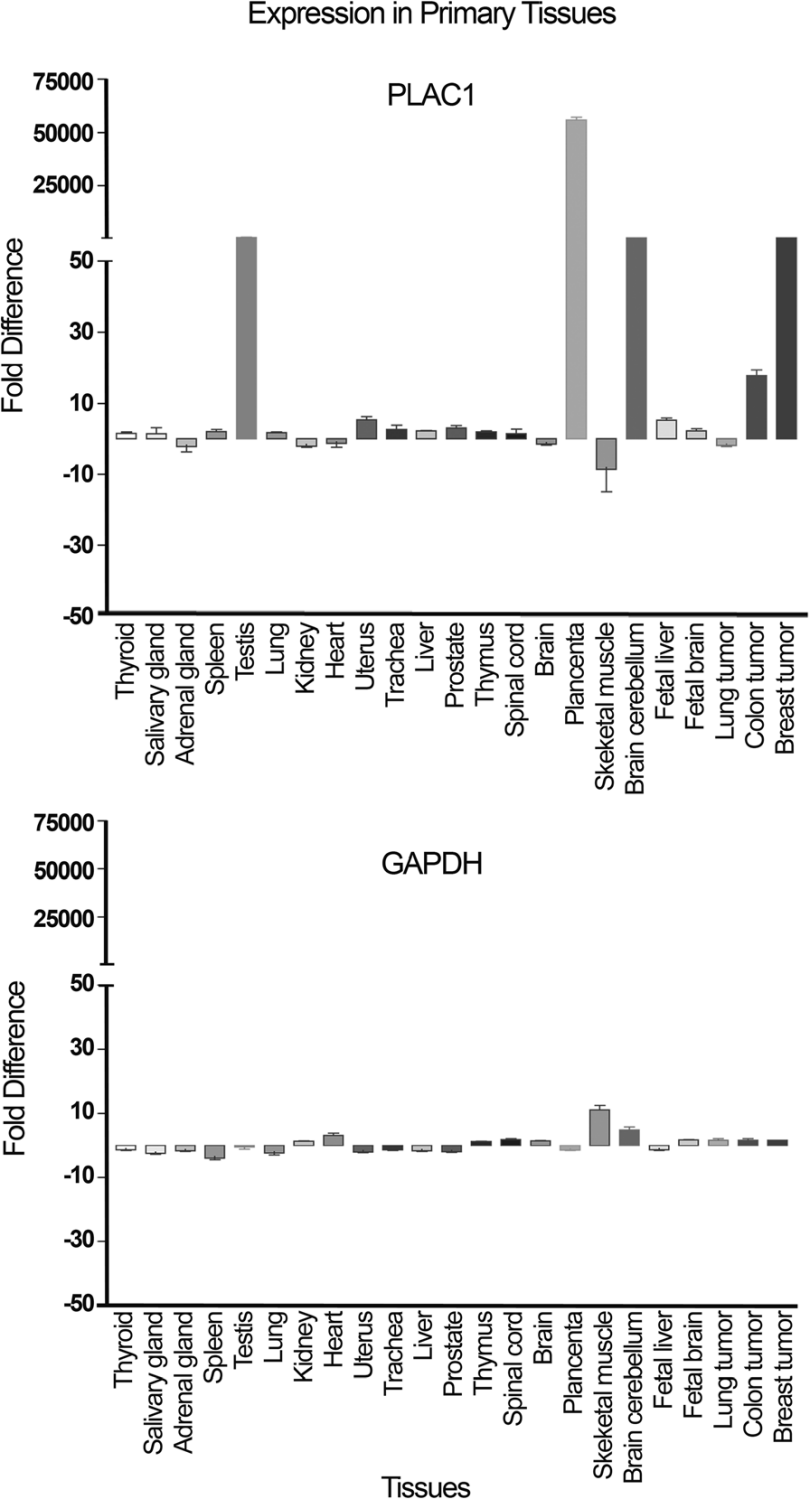
**Analysis of PLAC1 expression in RNAs derived from primary tissues and selected primary tumor tissues.** Real time RT-PCR was carried out using primer set 1. Fold difference is the expression level relative to the average of expression in tissues with low expression (i.e. all normal tissues except testis, placenta and brain cerebellum).

Previous studies had shown that expression of PLAC1 in normal tissues is restricted to placenta [1,2] by Northern blot analysis. We assessed a panel of RNA isolated from 20 primary tissues using a more sensitive technique (quantitative RT-PCR) to more thoroughly address this issue. As shown in Figure 3, little or no expression was observed in most of the tissues tested while robust expression was observed in RNA isolated from placenta. Nevertheless, this analysis also revealed expression in RNA isolated from testis and brain cerebellum. Analysis of PLAC1 expression in selected primary tumor tissues revealed low expression in individual lung and colon tumors but considerably higher expression in a breast tumor (Figure 3).

**Figure 4:**
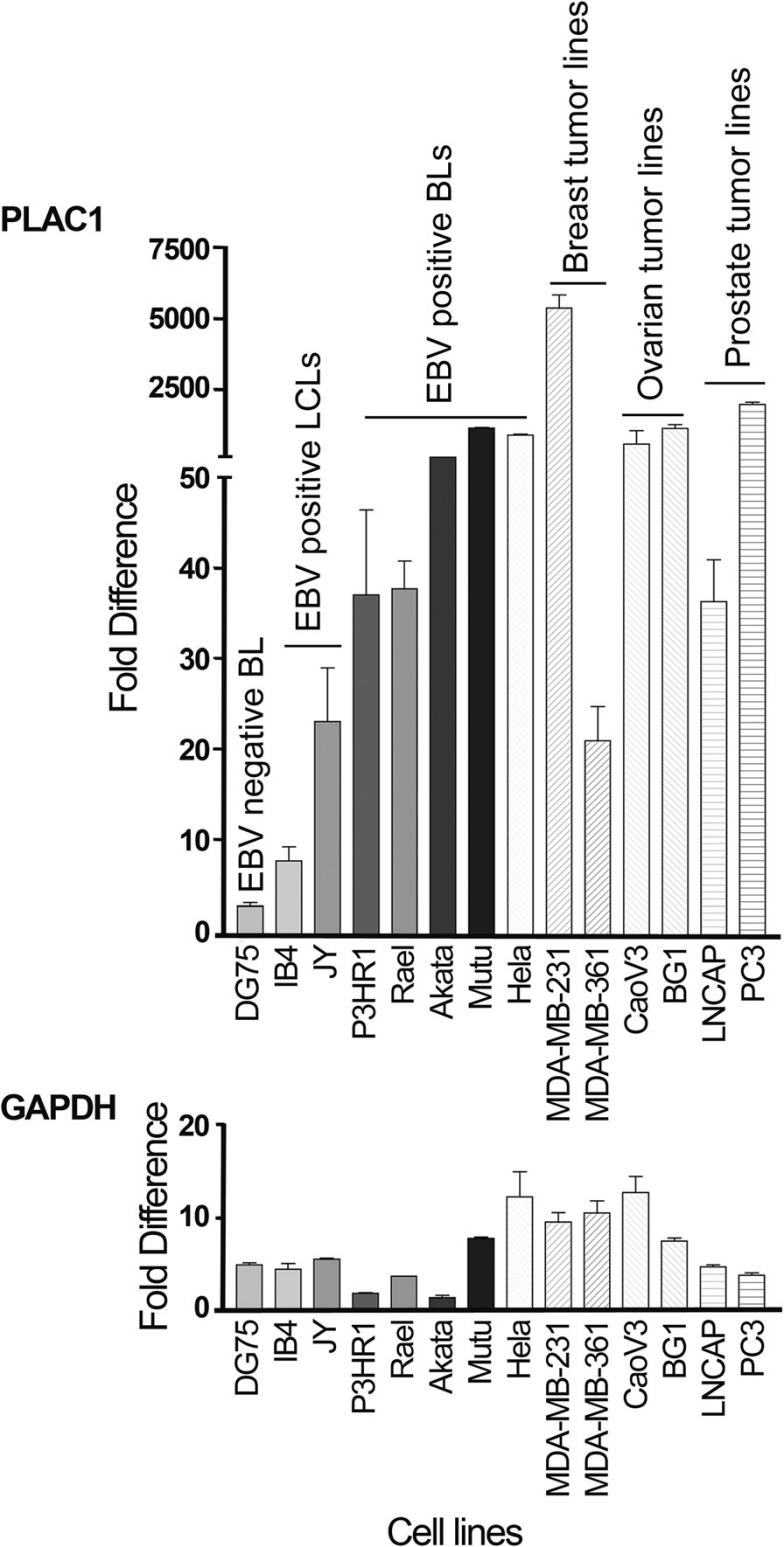
**Analysis of PLAC1 expression in various tumor and non-tumor derived cell lines.** DG75 is an EBV negative Burkitt’s lymphoma cell line. IB4 and JY are EBV immortalized lymphoblastoid cell lines and P3HR1, Rael, Akata, and MutuI are EBV positive Burkitt’s lymphoma cell lines. MDA-MB-231 and MDA-MB-361 are breast cancer derived cell lines, CaoV3 and BG1 are ovarian cancer derived cell lines, LNCAP and PC3 are prostate cancer derived cell lines. Fold difference represents the level of PLAC1 or GAPDH expression in the indicated cell lines vs. the average expression of normal tissues from Figure 3).

We next analyzed a panel of EBV infected non-tumor lymphomblastoid cell lines, EBV infected BLs, as well as cell lines derived from several different tumor types. As shown in Figure 4, the expression level of PLAC1 was significantly higher in four EBV positive Burkitt’s lymphoma cell lines compared with the EBV negative Burkitt’s lymphoma cell line, DG75. Some expression was observed in two EBV positive lymphoblastoid cell lines. High expression was observed in Hela cells and the two ovarian tumor cell lines tested (Figure 4). The highest expression was observed in the highly aggressive and invasive breast cancer cell line, MDA-MB-231 [21], whereas relatively lower expression was observed in the less aggressive breast tumor line, MD-MB-361. Expression was also observed in two prostate cancer cell lines tested while significantly higher expression was observed in the more aggressive androgen receptor negative line, PC3. These results indicate that PLAC1 is expressed in several EBV associated BL lines as well as in several tumor cell lines not associated with EBV.

**Figure 5:**
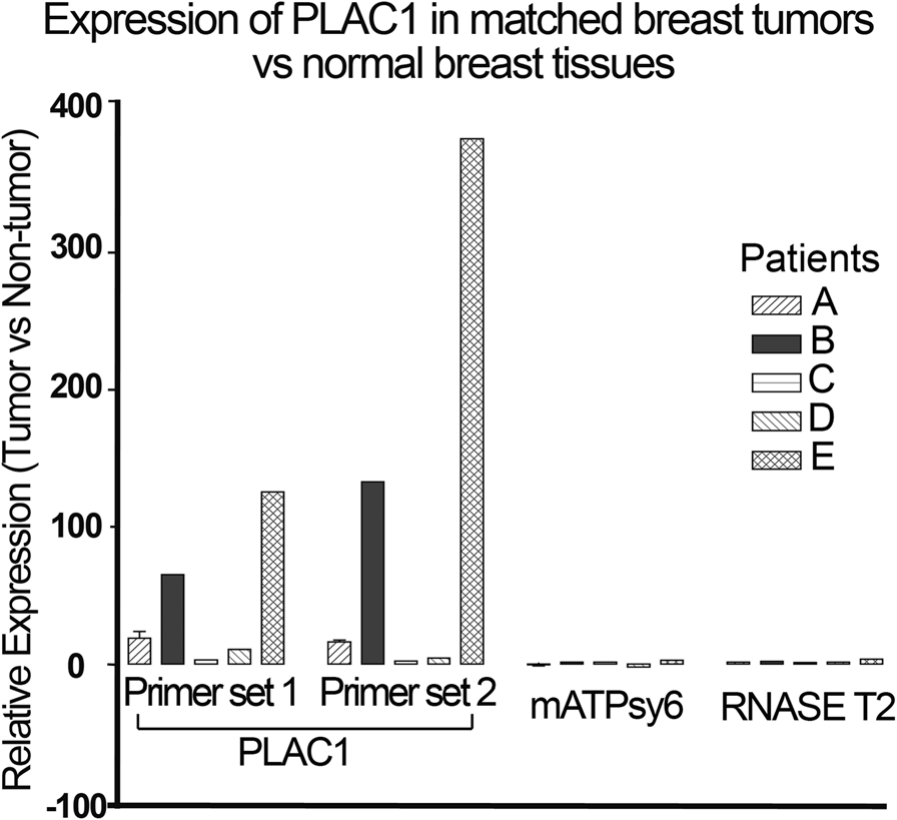
**Comparison of PLAC1 expression in matched primary tissue from breast cancer patients.** RNAs from tumor or adjacent normal tissue were analyzed by real time RT-PCR using primer set 1, primer set 2 and the two control genes, mATPsy6 and RNASE T2. Values represent expression in tumor RNA vs. corresponding normal adjacent tissue. GAPDH was excluded as a control because previous studies have shown a correlation between GAPDH expression and breast cancer aggressiveness [23,24].

We next assessed PLAC1 expression in matched tissues from breast cancer patients comparing expression in the tumor mass relative to adjacent non-tumor tissue. As shown in Figure 5, significantly higher expression was observed in tumor vs. normal breast tissue in patients B and E. In contrast, little difference in the expression of the housekeeping genes, mATPsy6 [22] and RNASE T2 was observed. It will be interesting to determine the status of EBV in these primary breast tumors though the matched DNA is not available for these samples.

## Discussion

We show here that like previous studies [1,2], PLAC1 expression is highly limited in normal tissues. Nevertheless, using real time RT-PCR, we have observed that PLAC1 expression can be detected in testis and cerebellum. PLAC1’s higher level of expression in EBV positive MutuI cells relative to EBV negative Mutu cells led us to test the expression PLAC1 in a panel of tumor cell lines. This analysis revealed that PLAC1 is highly expressed in several Burkitt’s lymphoma cell lines as well as tumor cell lines derived from a number of other cancers. Using RNAs isolated from primary matched tumor and normal tissues, we further determined a higher level of PLAC1 expression is some tumor samples. This raises the possibility that PLAC1 may be a member of the expanding group of CT antigens.

The first CT antigens described were identified using screens to identify antigenic proteins in cancer patients that are specifically expressed in tumor cells with the hopes of identifying targets for immunotherapy. Another common but not exclusive feature of CT antigens is their inducibility by hypomethylating agents and/or histone deacetylase inhibitors. At this time we have not tested this issue but it is notable that the promoter for PLAC1 is devoid of CpG dinucleotides and therefore may not be subjected to CpG methylation dependent silencing. Nevertheless, it does seem likely that PLAC1 might be silenced through some epigenetic imprinting mechanism involving histone deacetylases.

There are several functions that both the placenta and tumors provide which are required for and/or enhance the survival of the fetus and the tumor. The placenta is the key organ responsible for the exchange of nutrients and waste materials to and from the fetus. While these activities are mediated by specific transport mechanisms, this process is enhanced through the synthesis of angiogenesis promoting factors such as vascular endothelial growth factor (VEGF) and human chorionic gonadotropin (hCG) by the trophoblast [6,7]. Similarly, hypoxic conditions in tumors can promote the synthesis of angiogenic factors such as VEGF, which in turn leads to angiogenesis. Another key function of the placenta is to protect the fetus from immuno-surveillance by the mother. Both the fetus and the placenta are of fetal origin and therefore are immunologically distinct from the mother. The placenta utilizes multiple mechanisms to prevent immunological targeting by the mother’s immune system including the down regulation of major histocompatibility complex (MHC) II proteins [25]. Similarly, tumors have been shown to down regulate MHC proteins [26]. Tumors can also secrete cytokines that enhance the recruitment of tolerance inducing immune cells [27] and this also appears to be a key mechanism utilized by the placenta to prevent fetal targeting [28]. Another commonality between the synciotrophoblasts and invasive tumors is their production of metalloproteinases (MMPs) which serve to promote the invasive properties of the tumor and of the synciotrophoblasts [4].

Other studies have previously found molecular links between trophoblasts and malignant cells. Acevedo et al. discovered that 28 out of 28 randomly selected cancer cell lines express a membrane form of hCG [29]. In another study, Goshen et al. found that a specific splice variant of CD44 which is found in metastasizing human malignancies and is thought to play an important role in metastatic spread, is also expressed in invasive extravillous trophoblasts [30]. These authors propose that this splice variant may similarly play a role in trophoblast invasion. Interestingly, a previous study also found that another Epstein-Barr virus induced gene, EBI3 (Epstein-Barr virus inducible 3) is expressed at high levels in the syncytiotrophoblasts [31,32]. Together, there is accumulating data indicating that there are not only numerous functional similarities between tumors and the placenta but that there are also some similarities in the mechanisms, which mediate these common functions. The findings that PLAC1 is expressed in both tumors and in trophoblasts provide an additional molecular link between the two. While there is a limited knowledge regarding the function of PLAC1, there is circumstantial evidence indicating that it is crucial for fetal development [8] and for embryonic development [9]. Knockdown of PLAC1 in breast cancer cells not only blocks cell cycle and proliferation, but also impairs cell motility, migration and invasion probably through inhibiting the cyclin D1 and the phosphorylation of AKT kinase [11]. It will be interesting to ultimately determine how PLAC1 expression functions and whether it similarly promotes one or more key survival functions in tumors.

The number of latency associated genes expressed in EBV associated tissues varies according to the tumor and cell type. Typically, primary Burkitt’s lymphomas express a limited set of viral genes: EBNA1, two small non-coding Pol III derived transcripts referred to as EBER1 and EBER2, and a set of alternatively spliced products derived from the BamHI A region of the EBV genome (latency type I genes) [17]. In EBV immortalized non-tumor lymphoblastoid cell lines, a larger group of EBV encoded genes are expressed (latency type III genes) which are important for maintaining the growth phenotype of the infected cell [17]. In this case, the EBV encoded genes, EBNA1, EBERs, BamHI transcripts, plus EBNA2, EBNA-LP, EBNA3A, EBNA3B, EBNA3C and the latent membrane proteins, LMP1 and LMP2 are expressed. Many of the EBV genes unique to the type III program (relative to the type I program) are each essential for providing the growth phenotype to lymphoblastoid cell lines. In line with this, the growth phenotype of Burkitt’s lymphoma cells is probably dictated principally through genetic alterations such as the c-Myc translocation to the immunoglobulin locus [33], the defining translocation of Burkitt’s lymphomas. Accordingly, the EBV genome can be eradicated in Burkitt’s lymphoma cell lines without abrogating the proliferative capacity of the derived cells [18-20]. Nevertheless, it is clear that type I gene expression contributes to the tumor phenotype of Burkitt’s lymphoma cells since the loss of EBV in these cells results in cells that cannot form colonies in soft agar and cannot form tumors in SCID mice. This indicates that the type I genes likely play key roles in tumorigenesis other than simply promoting cell growth and it is possible that type I EBV gene expression contributes some of the characteristics that are common to tumors and placenta outlined above. It is notable that of the B-cell lines tested, the highest expression of PLAC1 was observed in the type I BL lines (note: although P3HR1 is a Burkitt’s lymphoma derived cell line, these cells display a group II/III gene expression pattern). This indicates that induction of PLAC1 by EBV is likely facilitated principally by the type I gene expression pattern. It will be interesting to determine whether expression of PLAC1 might contribute to one or more of the tumorigenic properties of EBV.

## Conclusions

Enforced genome loss of EBV and carried out gene array and real time RT-PCR analysis, we identify that EBV induces PLAC1 gene. Based on its expression profile and its localization to the X chromosome, we propose that PLAC1 is a candidate CT antigen.

## Materials and methods

### Cell culture

All B-cell lines, LNCAP and PC3 cell lines were propagated in RPMI supplemented with 10% fetal bovine serum albumin, penicillin, streptomycin, and glutamine. Hela, 293, MDA-MB-231, MDA-MD-361, CaoV3, and BG1 cells were propagated in Dulbecco’s Modified Eagles Medium (DMEM; Life Technologies) supplemented with 10% fetal bovine serum albumin, penicillin, streptomycin, and glutamine. Cells were cultured at 37°C in a humidified 5% CO_2_-containing atmosphere.

### Retroviral infections

Transient transfection experiments were performed by using a modified version of the calcium phosphate precipitation procedure (a detailed protocol is available at flemingtonlab.com). Briefly, 10^6^ cells were plated onto 100-mm-diameter tissue culture dishes. The following day, the medium was replaced with 8 ml of fresh supplemented DMEM; 4 h later, DNA precipitates were generated by mixing 0.5 ml of 1x HEPES-buffered saline (0.5% HEPES, 0.8% NaCl, 0.1% dextrose, 0.01% anhydrous Na_2_HPO_4_, 0.37% KCl [pH 7.10]) with a total of 30 μg of plasmid DNA (10 μg retroviral vector plus 10 μg VSV-G expression vector, plus 10 μg pVPACK dGI packaging vector). A total of 30 μl of 2.5 M CaCl_2_ was added, and samples were mixed immediately. Precipitates were allowed to form at room temperature for 20 min before being added drop wise to cells. Cells were incubated at 37°C with 5% CO2 for 16 h before the medium was replaced with 10 ml of fresh DMEM (plus 10% FBS).

24 hrs later, viral supernatants were collected and subjected to two rounds of centrifugation to get rid of floating 293 cells. Infections were carried out in 6 well plates with 1 ml virus plus 10^6^ MutuI or DG75 cells suspended in 1 ml DME (+10% fetal bovine serum). Polybrene was added to a final concentration of 8 ug/ml and the mixture was mixed by gently rocking. Cells were spun in 6 well plates at 1000 g for 1 hr followed by 4 hr incubation in a tissue culture incubator. Cells were then collected, spun down and resuspended in 2 ml RPMI (+10% fetal bovine serum, + penicillin, streptomycin, and glutamine) per well. Cells were cultured for 2 days prior to antibiotic selection.

### Western blot analysis

After a single 1× PBS wash, cells were immediately suspended in 300 μl of sodium dodecyl sulfate (SDS)-polyacrylamide gel electrophoresis loading buffer (125 mM Tris [pH 6.80], 10% glycerol, 2% SDS, 5% 2-mercaptoethanol, 0.05% bromphenol blue) and boiled for 20 min to shear the genomic DNA. Whole-cell extracts were measured with the Bio-Rad protein assay kit according to the manufacturer's instructions. An equal weight of cell lysates was subjected to SDS-polyacrylamide gel electrophoresis and transferred to nitrocellulose membranes. The blots were blocked for 30 min in Tris-buffered saline containing 5% low-fat powdered milk and 1% FBS and then incubated with the primary antibody (in blocking buffer) overnight at 4°C. The blots were washed three times with 1× TBST (140 mM NaCl, 3 mM KCl, 25 mM Tris–HCl [pH 7.4], 0.1% Tween 20) (each wash was carried out for approximately 10 min). The blots were then incubated with horseradish peroxidase-conjugated secondary antibody (Bio-Rad) in blocking buffer for 1 h at room temperature. Blots were washed as described above and analyzed with an enhanced chemiluminescence detection system (Perkin-Elmer) according to the manufacturer's recommendations, and filters were exposed to Fuji Super RX film. The following antibodies were used for Western blot analysis: EBNA1 (Advanced Biotechonologies Incorporated, CAT# 13-156-100, 1:1000 dilution), HA.11 (Boehringer Mannheim, Cat# 1 867 431, 1:1000 dilution), Actin (Sigma, Cat# A 4700, 1:1000 dilution).

### Real time RT-PCR

RNAs from cells were prepared using a RNeasy® Mini kit from Qiagen (Cat# 74104) according to vendor’s protocol. Normal tissue RNAs were purchased from BD Biosciences (Cat# 636643). Individual RNAs from primary lung (Cat#64013-1), colon (Cat#64014-1), and breast (Cat#64015-1) tumors were purchases from BD Biosciences. qPCR human matched pair total RNA panel was purchased from BD Biosciences (Cat# 636691).

cDNA synthesis was carried out using Superscript™ III first-strand synthesis system for RT-PCR (Invitrogen, Cat#. 18080–051). 2 ug of RNA were used for first strand synthesis and then diluted to a final volume of 200 ul.

Real Time PCR was carried out using 5 ul of diluted cDNA for each 20 ul reaction. Analysis was carried out on a BioRad iCycler using BioRad iQ™ SYBR Green supermix (CAT#170-8882) with the following conditions: 95°C 3' then 40 cycles of 95°C 30", 60°C 30", and 72C 30”. Primer sequences for real time PCR were the following: PLAC1; PLAC1-PS1-sense, 5' - TTCACCAGTGAGCACAAAGC - 3' and PLAC1-PS1-antisense, 5' - CCAGTCTATGGAGCACAGCA - 3', PLAC1-PS2-sense 5' -GGGCAAATACAGACACAGCA -3' and PLAC1-PS2-antisense 5' -CCAGTCTATGGAGCACAGCA -3', GAPDH; GAPDH-sense, 5' - GCCAAGGTCATCCATGACAACTTTGG - 3' and GAPDH-antisense, 5' - GCCTGCTTCACCACCTTCTTGATGTC - 3', mATPsy6 [22]; mATGsy6-sense, 5’-GGGCGCAGTGATTATAGGCTT-3’ and mATPsy6-antisense, 5’-GGTGTAGGTGTGCCTTGTGGT-3’, RNASE T2; RNASE T2-sense, 5' -TGGGGATAAAACCATCCATC -3' and RNASE T2-antisense, 5'-AGCTGCTGGTCTTGCTTAGT-3'.

For analysis of EBV DNA in Mutu Clones, genomic DNA was isolated as describe at flemingtonlab.com. PCR analysis was carried out essentially as described above for RT-PCR analysis except that the following conditions were used: 95°C 3' then 40 cycles of 95°C 30", 55°C 30", and 72C 30”. The following primers were used to amplify different parts of the EBV genome: BamHI R primers (Rp); R-left, 5'-TAGTTAATGCCCCAGCCAGA-3', R-right, 5'-CTTTAAAAAGGCCGGCTGAC-3', BamHI M primers (BMRF1p) [34]; M-right, 5'-ACCTACATGACTAGCATCAAGCAA-3', and M-left, 5'-GGCCTCCATAGTTTACAGACAGAA-3', BamHI Q primers (Qp); Q-left, 5'-AAATTGGGTGACCACTGAGG-3' and Q-right, 5'-CATACACCGTGCGAAAAGAA-3', BamHI K primers (EBNA1); K-left, 5'-AAGGAGGGTGGTTTGGAAAG-3' and K-right, 5'-TGGAATAGCAAGGCCAATTCC-3'.

### Plasmid construction

To generate dominant negative forms of EBNA1, full length EBNA1 was first excised from the plasmid, pCEP4, with ClaI and SacII. Since the SacII digestion cleaves the last few carboxy-terminal amino acids from the EBNA1 reading frame, two adaptors were synthesized which together encode the terminal amino acids from EBNA1 plus a BglII overhang (Adaptor 1-sense; 5’-GGAGGGTGATGACGGAGATGACGGAGATGAA-3’, Adaptor 1-antisense; 5’-CATCACCTCCTTCATCTCCGTCATCTCCGTCATCACCCTCCGC-3’, Adaptor 2-sense; 5’-GGAGGTGATGGAGATGAGGGTGAGGAAGGGCAGGAGTGA-3’, Adaptor 2-antisense; 5’-GATCTCACTCCTGCCCTTCCTCACCCTCATCTC-3’). The ClaI/SacII EBNA1 fragment was ligated to EcoRI and BglII cut pMSCV-neo vector in the presence of Adaptor 1 plus Adaptor 2, plus an EcoRI/ClaI adaptor (from eZclone Systems). Ligations were carried out in the presence of 1 ul T4 polynucleotide Kinase to phosphorylate adaptors during ligation reaction. This construction led to the generation of pMSCV-neo-EBNA1wt. pMSCV-neo-E1dn was generated from this plasmid by digesting pMSCV-neo-EBNA1wt with EcoRI and ApaI to excise the amino terminal EBNA1 sequences. The resulting vector plus carboxyl terminal EBNA1 sequences were then ligated to three adaptors which when linked together contain a translation initiation sequence, an HA epitope tag, the SV40 nuclear localization signal plus an EcoRI overhang at the 5’ end and an ApaI overhang at the 3’ end (DN adaptor 1-sense; 5’-AATTCTGCTGAAGATGATGGCCTATCCTTATG-3’, DN adaptor 1-antisense; 5’-CATCATCTTCAGCAG-3’, DN adaptor 2-sense; 5’-ATGTGCCTGACTATGCCGCCCCAAAGAAA-3’, DN adaptor 2-antisense; 5’-CATAGTCAGGCACATCATAAGGATAGGC-3’ , DN adaptor 3-sense; 5’-AAGCGAAAGGTGGCCGGCC-‘3, DN adaptor 3-antisense; 5’-GGCCACCTTTCGCTTTTTCTTTGGGGCGG-3’). Ligations were carried out in a standard ligation reaction except that 1 ul of T4 polynucleotide kinase was added to phosphorylate adaptors during ligation reaction. This strategy generated pMSCV-neo-EBNA1dn. pMSCV-puro-EBNA1dn was derived from this plasmid by simply excising the HA/nls/EBNA1dn cassette from pMSCV-neo-EBNA1dn and transferring to pMSCV-puro using appropriate standard cloning adaptors. All constructs were verified by sequence analysis.

## Competing interests

The authors declare that they have no competing interests.

## Authors’ contributions

XW designed and conducted real time RT-PCR experiments. MB performed cell lysate extraction and maintained cell lines. QY analyzed data and wrote the manuscript. All authors read and approved the final manuscript.
